# Editorial: Immune evasion mechanisms and their role in the pathogenesis of autoimmune disorders

**DOI:** 10.3389/fimmu.2023.1267922

**Published:** 2023-09-13

**Authors:** Fabiana Rizzo, Gunnar Houen

**Affiliations:** ^1^ Department of Infectious Diseases, Istituto Superiore di Sanità, Rome, Italy; ^2^ Department of Neurology, Rigshospitalet, Research Park, Glostrup, Denmark

**Keywords:** apoptosis, EBV, autoimmunity, disorders, drugs

Autoimmune diseases result from a combination of genetic and environmental factors that disrupt normal immune responses to infections while also preventing autoimmune reactions (1, 2). Genes governing antigen detection, processing, and lymphocyte interactions play a crucial role among genetic factors (2). Among environmental factors, infections and influences on immune system function are particularly significant (2). The Epstein-Barr Virus (EBV) stands out, associated with various autoimmune diseases alongside its role in certain cancers (2–5). This association is supported by epidemiological data and an understanding of EBV’s immune evasion mechanisms (3, 5).

Within this Research Topic, Sharifinejad et al. analyze patients with monogenic combined immunodeficiencies, revealing autoimmune manifestations in about 18% of cases, often linked to mutations in immune system genes, highlighting the intricate link between immune deficiencies and autoimmunity.

Schönrich et al. discuss the central role of EBV in multiple sclerosis, focusing on the virally encoded IL-10 homologue and its impact on host IL-10 production, illustrating a significant immune evasion mechanism and its implications for central nervous system pathology.

The connection between immune system genes, infections, and autoimmunity is further explored by Xiao et al. in a meta-analysis of IFIH1 polymorphisms, showing positive correlations with type 1 diabetes, systemic lupus erythematosus, and multiple sclerosis.

Primary Sjögren’s Syndrome, a systemic autoimmune disease, is associated with B cell hyperactivity, lymphocyte infiltration, and destruction of exocrine glands, with potential links to EBV infection (3, 6). Hinrichs et al. demonstrate that SS patients exhibit distinct mucosa-associated invariant T (MAIT) cell behavior, expressing higher levels of certain markers that facilitate exocrine gland infiltration and pathology.

Dermatomyositis (DM), characterized by muscle weakness and rashes, may also be connected to immunodeficiency and infections (7). Hilliard et al. show alterations in the natural killer (NK) cell population in juvenile DM, which could indicate impaired NK cell function.

Collectively, these articles underscore the intricate relationship between immunodeficiency, viral immune evasion mechanisms, and autoimmune conditions, offering valuable insights for the treatment and prevention of such diseases.

## Author contributions

FR: Conceptualization, Resources, Visualization, Writing – review & editing, Methodology, Investigation. GH: Visualization, conceptualization, Writing – review & editing.
